# Our New York Letter

**Published:** 1889-04

**Authors:** M. B. Hutchins

**Affiliations:** New York


### Correspondence.

### OUR NEW YORK LETTER.

### DERMATOLOGICAL NOTES—ANTISEPTICS.

*Editors Atlanta Medical and Surgical Journal:*

A rather peculiar hypertrophic condition was noticed in a young woman who came to the Skin and Cancer Hospital for treatment. The left ear was twice as large as the right, and covered with a thick growth of hair. The lobe was especially noticeable for its thickness, and the whole ear was of a dirty livid color. The trouble was congenital. No treatment was ordered.

Some one has recommended the following as a good adhesive and antiseptic ointment: ℞: Ceræ Flavæ, 40 parts; Lanolin, 40; Olei Olivæ, 17; Acid. Salicyl , 3. M.

Recently, out of New York, I saw simple powder of oxide of zinc used as antiseptic dressing after a cutaneous operation. The wound healed "by first intention." Here we find as great difference among surgeons in regard to antisepsis as anywhere. The more "advanced" school are particularly strict in its employment. The older surgeons, many of them, are content with rendering implements aseptic, and using antiseptic dressings afterwards. The younger surgeons are the most particular as a general thing in the operations which I have seen.

Dr. Bulkley is pleased with the effect of prescriptions containing fluid ext. rumex (yellow dock), but seems to admit that its use is empirical. Considered beneficial in a number of cases requiring a tonic.

A boy, aged sixteen, came to the clinic the other day, who has the most complete alopecia areata (or what Crocker, of

England, would call, alopecia areata universal), that I have ever seen. The scalp was completely bald, save here and there a solitary hair. A dermatologist says the disease may as easily be cured with *distilled water*, as with the most powerful irritants. In other words, recovery may take place at any time spontaneously.

One objection to the plasters, mentioned in my last letter, is that the adhesive material sometimes produces irritation, evidenced by the appearance of small points denuded of epidermis and inclined to formation of scales.

Duhring, says the eruption from *belladonna* is one of those most frequently encountered, among the drug eruptions. A beautiful example was at the clinic, in the person of a child eight or nine years old, whose physician had prescribed belladonna for some "trouble." The neck, arms and shoulders were covered with a bright erythema, and the throat and tongue somewhat irritated. Upon inspection of the erythematous skin, numerous dilated capillaries were seen—in groups. Many of these showed a purplish color, and this color predominated in the groups.

A Swedish woman, aged sixty, of splendid physique, was admitted to the hospital two weeks ago considerably "broken in health" from cancer of the womb. She was put on treatment to build her up and to-day the uterus was removed entire as so much was diseased as to render the operation justifiable. The operation was performed by Dr. Jauvrin. The patient was etherized and the knees drawn well back towards the abdomen. A large retractor was then used to draw down the perineum and a loop of surgeon's silk passed by a needle through the anterior part of cervix—this portion being sound. The uterus was then drawn downward, the retractor previously introduced withdrawn, and one introduced on either side. The vaginal attachment of cervix then divided with curved scissors anteriorly. The handle of a tenaculum was used when possible. When the anterior portion of cervix had been separated from the tissues between it and the bladder, the posterior wall was similarly divided. An opening was then made into the peritoneal cavity.

Capillary hemorrhage was checked by the use of the hot water douche. The uterus was then drawn forward and downward into the vagina. The broad ligament of the right sight was freed from adjacent tissue close to the uterus, and clamped with long-bladed forceps, two being necessary to completely include the ligament. The ligament was then separated from the uterus by means of the Paquelin cautery. Ligament of left side similarly treated and the uterus removed. The clamping forceps were allowed to remain, the vagina was well washed out with hot water and packed with strips of iodoform gauze. There was no shock, and the patient was doing well when last seen to-day. During a part of the operation a sound was kept in the bladder as a guide.

M. B. HUTCHINS, M. D.

*New York, March 1889.*

---

THE DIRECT APPLICATION OF COPAIBA IN GONORRHŒA.—M. Rively has treated nine cases of gonorrhœa in the first stage by balsam of copaiba applied locally. He introduced a bougie containing the balsam into the urethra as far as the membranous portion, and allowed it to remain from six to eight minutes. Applications were thus made once daily for three or four days. In eight of the nine cases pain in micturition rapidly disappeared, and after a day or so the discharge ceased. In the ninth case the result was less favorable, owing to the patient continuing sexual intercourse and drinking large quantities of beer. No success followed the treatment of gleet by this method.—*Med. Register.*

---

BETTER THAN NOTHING.—A very handsome man, of large form, fell off the railway platform at Camp Hill, falling under the train, and had one thigh and the leg of the other below the knee completely smashed. He was brought to the General Hospital, and both limbs were at once amputated. The case gave me much anxiety; but he continued to live, the wounds healing well. I found he was engaged to be married to a very nice, respectable young woman, whom I found one evening sitting by his side. I said I thought she should think, very seriously about her engagement, hinting that I thought it had better be broken off; but she most naively said, "Oh! sir, I am quite content to take the rest of him."—Mr. Dickinson Copton, in *Guy's Hospital Reports.*

